# Development of Xanthoangelol-Derived Compounds with Membrane-Disrupting Effects against Gram-Positive Bacteria

**DOI:** 10.3390/antibiotics13080744

**Published:** 2024-08-07

**Authors:** Siyu Yang, Fangquan Liu, Yue Leng, Meiyue Zhang, Lei Zhang, Xuekun Wang, Yinhu Wang

**Affiliations:** State Key Laboratory for Macromolecule Drugs and Large-Scale Manufacturing, School of Pharmaceutical Sciences and Food Engineering, Liaocheng University, Liaocheng 252059, China; 17861827217@163.com (S.Y.); a13188742050@163.com (F.L.); lengyue0918@126.com (Y.L.); 17862567273@163.com (M.Z.); 17862567321@163.com (L.Z.)

**Keywords:** bacterial resistance, antibacterial agents, mimics of AMPs, membrane-disrupting

## Abstract

Infections caused by multidrug-resistant pathogens have emerged as a serious threat to public health. To develop new antibacterial agents to combat such drug-resistant bacteria, a class of novel amphiphilic xanthoangelol-derived compounds were designed and synthesized by mimicking the structure and function of antimicrobial peptides (AMPs). Among them, compound **9h** displayed excellent antimicrobial activity against the Gram-positive strains tested (MICs = 0.5–2 μg/mL), comparable to vancomycin, and with low hemolytic toxicity and good membrane selectivity. Additionally, compound **9h** demonstrated rapid bactericidal effects, low resistance frequency, low cytotoxicity, and good plasma stability. Mechanistic studies further revealed that compound **9h** had good membrane-targeting ability and was able to destroy the integrity of bacterial cell membranes, causing an increase in intracellular ROS and the leakage of DNA and proteins, thus accelerating bacterial death. These results make **9h** a promising antimicrobial candidate to combat bacterial infection.

## 1. Introduction

The emergence and spread of multidrug-resistant bacteria pose a significant threat to global public health, leading to increased morbidity and mortality [1,2,3]. In particular, the most worrying ESKAPE pathogens, namely, *Enterococcus faecium*, *Staphylococcus aureus*, *Klebsiella pneumoniae*, *Acinetobacter baumannii*, *Pseudomonas aeruginosa*, and *Enterobacteriaceae*, have limited the efficacy of most of frontline antibiotics as they have developed resistance to most of the conventional antibiotics [4,5,6]. In addition, the decreasing investment from pharmaceutical companies in the discovery and development of new antibacterial agents has led to a continuous decline in the number of new antibiotic approvals, which further exacerbates the incidence of antibacterial resistance [7,8,9]. Currently, almost 700,000 people die from bacterial infections each year, and the deaths are expected to rise to 10 million annually by 2050 if current trends continue [10,11]. The alarmingly spread of MDR pathogens, alongside the decline in the effectiveness of existing antibiotics, has compelled researchers to look for new alternatives with unique mechanisms of action to address antimicrobial resistance. 

**Figure 1:**
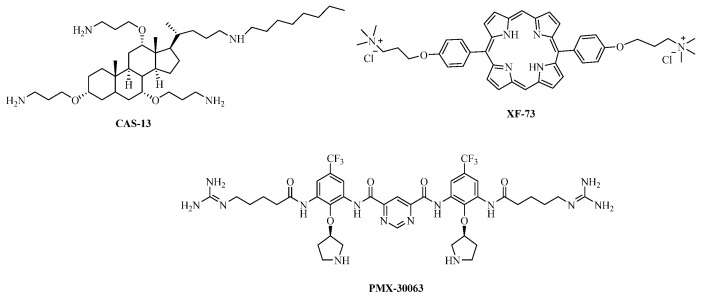
Structure of CAS-13, XF-73, and PMX-30063.

Antimicrobial peptides (AMPs), also called host-defense peptides, have been used by almost all multicellular organisms as an initial defense mechanism against pathogenic microbes [12,13]. AMPs are typically short peptides consisting of 10–50 amino acids [14]. Despite a wide range of structural diversity in terms of sequence and secondary structure, these AMPs share common characteristics: being amphipathic in nature, with net cationic charge [15,16,17]. This amphipathic nature of AMPs is believed to be the structural basis for their activity, and the bacterial membrane is considered as the main target for AMPs [18,19]. The cationic groups in AMPs play an essential role for their initial attachment to the negatively charged bacterial membranes via electrostatic attraction, which is followed by the interaction of the hydrophobic side chains of AMPs with the lipid-rich cytoplasmic membrane, causing the disruption of membrane integrity, and ultimately causing cell death [20,21]. Unlike conventional antibiotics, AMPs offer several advantages as potential antibacterial agents as they exhibit rapid and broad-spectrum antibacterial activity, reduced susceptibility to resistance, high selectivity towards microbial cells, and good synergy with other antimicrobials [22,23]. Although natural AMPs show potential as innovative antimicrobials, their clinical development has been hindered by challenges such as poor bioavailability, low proteolytic stability, unknown in vivo toxicity, and high production costs [24,25]. To address these challenges, researchers have increasingly focused on the development of non-natural small-molecule antimicrobial peptidomimetics. These peptidomimetics can be defined as compounds bearing unnatural backbones that mimic the amphipathic structure and biological function of the natural AMPs [26,27]. These small-molecule peptidomimetics can surmount the limitations associated with natural AMPs and display improved metabolic stability while retaining the similar activity and selectivity properties of AMPs. Their recent success is highlighted by three peptidomimetics (CSA-13, XF-73, and PMX-30063; Figure 1) in clinical trials [28,29,30,31]. 

**Figure 2:**
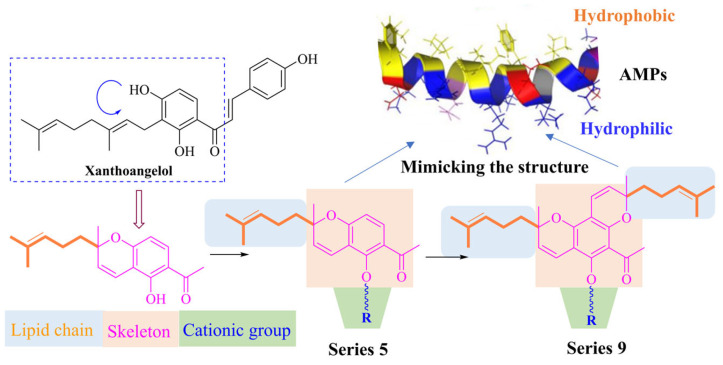
Design concept for amphiphilic xanthoangelol-derived compounds by mimicking the structure and the biological function of AMPs.

Natural products are a fascinating resource for drug development due to their structural diversity, offering a wide range of new scaffolds for antimicrobials and other bioactive components. Xanthoangelol, a natural geranylated hydroxy chalcone, is isolated from the roots of *Angelica keiskei*, and it has shown diverse therapeutic and biological properties, including anticancer [32,33], anti-inflammatory [34,35], and anti-microbial activities [36,37]. Recent research has shown that xanthoangelol exhibited good antibacterial activity against Gram-positive bacteria by destroying cell membranes, a mechanism similar to that of AMPs [38]. The geranyl chain of xanthoangelol meets the hydrophobic tail requirements observed in the structure of AMPs, facilitating its insertion into the lipid bilayer’s hydrophobic core. Nevertheless, xanthoangelol also has some drawbacks, such as poor water solubility, susceptibility to metabolism, and a narrow antibacterial spectrum. The hydrophobic chalcone scaffold and geranylgeranyl chain of xanthoangelol likely contribute to its poor water solubility, while the phenolic hydroxyl groups of xanthoangelol readily form covalent bonds with endogenous molecules like glucuronic acid or sulfuric acid, thereby increasing first-pass metabolism. Herein, to improve the water solubility, reduce the metabolic rate, and expand the antibacterial spectrum of xanthoangelol, we designed and synthesized a series of amphiphilic xanthoangelol-derived compounds as antimicrobials by mimicking the structure and function of AMPs. It has been widely realized that cationic charge, hydrophobicity, and molecular weight seem to be key structural parameters that control the antimicrobial potency and hemolytic activity of the antimicrobial peptidomimetics. The presence of highly cationic groups allows for adherence to the bacterial cell surfaces through electrostatic interactions; however, these groups are unlikely to penetrate the hydrophobic interior of the membrane, which restricts their activity [39]. On the other hand, peptidomimetics with excessive hydrophobicity can cause non-specific toxicity to both human and bacterial cells [40,41,42]. In addition, high molecular weights are detrimental to druggability. Therefore, it is crucial in the design of antimicrobial peptidomimetics to meticulously adjust the ratio of the cationic charge and hydrophobic characteristics, as well as to ensure a suitable molecular weight. Initially, we selected the left-hand-side structure of xanthoangelol for cyclization to obtain the pyranochromene scaffold (Figure 2); different cationic groups then were introduced on the scaffold to yield a range of amphipathic structures (Series 5). However, the resulting derivatives displayed only moderate antibacterial activity, which might have been due to the insufficient hydrophobic chain that failed to promote pore formation and cell death. Next, two lipophilic chains were incorporated into the pyranochromene scaffold to investigate their antibacterial activity (series 9). Compared with compounds in series 5, these compounds in series 9 showed significant improvement in the antibacterial activity. After the in vitro antibacterial evaluation of xanthoangelol-derived compounds, a promising compound **9h** with excellent antibacterial activity, low hemolytic activity, and good membrane selectivity was obtained. Plasma stability, bactericidal kinetics, drug resistance, and inhibition and disruption of bacterial biofilms and antibacterial mechanisms were also investigated. 

## 2. Results and Discussion

### 2.1. Chemistry

**Scheme 1:**
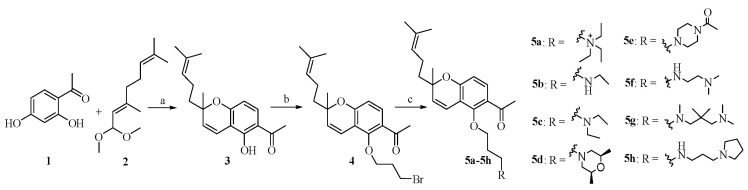
Reagents and conditions: (a) pyridine, 150 °C, 10 h; (b) K_2_CO_3_, 1,3-dibromopropane, CH_3_CN, 60 °C, 8 h; (c) RH, K_2_CO_3,_ CH_3_CN, 60 °C, 8 h.

**Scheme 2:**
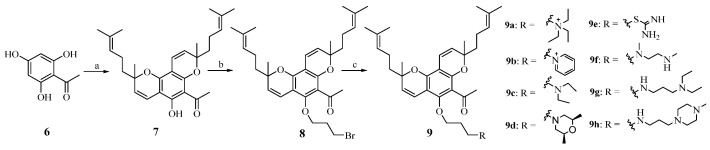
Reagents and conditions: (a) pyridine, 150 °C, 18 h; (b) K_2_CO_3_, CH_3_CN, 60 °C, 10 h; (c) RH, K_2_CO_3,_ CH_3_CN, rt, 8 h.

The synthetic routes of the target compounds are outlined in Scheme 1 and Scheme 2. Scheme 1 shows the synthetic route for compounds **5a**–**5h**. First, intermediate **3** was prepared by the cyclization reaction of commercial citraldimethylacetal **2** and ketone **1** in pyridine under reflux conditions. Then, intermediate **3** reacted with 1,3-dibromopropane in acetonitrile at 60 °C for 8 h to afford **4**. Finally, treatment of **4** with various amines obtained compounds **5a**–**5h**. 

Compounds **9a**–**9h** were synthesized using a similar method to that of compounds **5a**–**5h**, as illustrated in Scheme 2. The synthesis of intermediate **7**, in contrast to intermediate **3**, necessitated a higher quantity of citraldimethylacetal (4.0 eq) and a longer reaction time (18 h). Following this, compound **7** underwent alkylation with 1,3-dibromopropane and amination with different amines to yield compounds **9a**–**9h**.

### 2.2. In Vitro Antibacterial and Hemolytic Activity

All target derivatives were assessed for their in vitro antibacterial activities using a broth microdilution assay to test their minimum inhibitory concentrations (MICs) against six Gram-positive strains (*S. aureus* ATCC31007, *S. aureus* ATCC25923, *S. aureus* ATCC43300, *E. faecalis* ATCC29212, *B. subtilis* ATCC9372, and *S. epidermidis* ATCC12228) and four Gram-negative strains (*A. baumannii* ATCC19606, *K. pneumonia* ATCC10031, *E. coli* ATCC25922, and *P. aeruginosa* ATCC27853). These compounds were also evaluated for their hemolytic activities and membrane selectivity by determining their HC_50_ (the concentration required to lyse 50% of red blood cells) and SI (HC_50_/MICs) values, respectively, and vancomycin was used as the positive drug.

To begin with, compounds **5a**–**5h** consisting of an unsaturated lipid chain and a pyranochromene scaffold were synthesized and tested. Compounds **5a**–**5h** showed moderate or weak antibacterial activity against the eight Gram-positive bacterial strains tested (MICs ≥ 4 μg/mL) and poor antibacterial activity against the Gram-negative bacterial strain tested. As shown in Table 1 and Table 2, compounds **5a**–**5e** containing one tertiary amine or quaternary amine exhibited weak antimicrobial activity (MICs = 8–>128 μg/mL), while compound **5f**–**5h** containing two cationic amine groups showed moderate antibacterial activity against the Gram-positive strains tested (MICs = 8–32 μg/mL). This result suggests that incorporation of cationic groups was beneficial for improving the activity. Among them, compounds **5g** and **5h** were best, and they exhibited moderate activity against the three *S. aureus* tested (MICs = 8 μg/mL) and good activity against *S. epidermidis* ATCC12228 with an MIC value of 4 μg/mL. The unsatisfactory antibacterial performance in this series could be attributed to the insufficient hydrophobic chain that failed to insert into the phospholipid bilayer and promote pore formation. 

To further explore the effect of hydrophobicity from unsaturated lipid chains on antibacterial activity, pyranochromene derivatives with dual unsaturated chains (**9a**–**9h**) were synthesized. The results revealed that almost all derivatives in this series exhibited significantly improved antimicrobial properties against eight Gram-positive bacteria when compared to the compounds in series 5, which only possessed one unsaturated lipid chain. For example, compounds **5a** and **9a**, as well as **5c** and **9c**, shared the same scaffold and cationic group. The MICs against *S. aureus* ranged from 2 to 4 μg/mL for **9a** and **9c**, while compounds **5a** and **5c** exhibited weak antibacterial activity (MICs = 8–128 μg/mL), which further highlighted the importance of unsaturated lipid chains in enhancing activity. These derivatives in series 9, except **9d** and **9e**, displayed excellent antibacterial activity against the Gram-positive bacteria. For example, the MIC values of compound **9d** and **9e** against the eight tested positive bacteria were almost all higher than 64 μg/mL, suggesting their poor antimicrobial activity, while the MIC values of other compounds in this series 9 ranged from 0.5 to 32 μg/mL, indicating their potent antimicrobial properties. In particular, compound **9h** displayed the most potent antibacterial activity, demonstrating comparable activity (MICs = 0.5–8 μg/mL) to that of the positive control vancomycin against the Gram-positive bacteria. Additionally, it also exhibited good activity against the Gram-negative bacteria tested (MICs = 4–16 μg/mL). 

To further detect the hemolytic toxicity and therapeutic potential of these derivatives, we proceeded to examine the hemolytic activities and membrane selectivity of these derivatives. The HC_50_ value was defined as the concentration needed for a compound to lyse 50% of red blood cells, and it is often used to evaluate the hemolytic toxicity to eukaryotic cells. As shown in Table 1, compounds **5a**–**5h** showed poor hemolytic activities, with HC_50_ values of ranging from 62.3 to 746.5 μg/mL, suggesting that they were unlikely to lyse blood cells at MIC concentrations. In contrast, compounds in series 9 except for **9c**–**9e** showed moderate hemolytic activity with HC_50_ values of 26.6–57.9 μg/mL, indicating that these compounds have some hemolytic toxicity to eukaryotic cells. The selectivity index (SI) was calculated by HC_50_/MICs of *S. aureus* ATCC43300 and was used to assess the potency and safety of the compounds. As illustrated in Table 1, these xanthoangelol-derived compounds except for **9c** and **9h** possessed low membrane selectivity. Among these derivatives, compounds **9h** showed the most potent antibacterial activity with a MICs of 0.5–2 μg/mL against Gram-positive bacteria and exhibited low hemolytic toxicity (HC_50_ = 98.0 μg/mL) and the best membrane selectivity (SI = 49.0). Thus, compound **9h** was selected as a representative molecule for the in-depth in vitro and in vivo studies.

### 2.3. Plasma Stability and Bactericidal Activity in Mammalian Fluids

**Figure 3:**
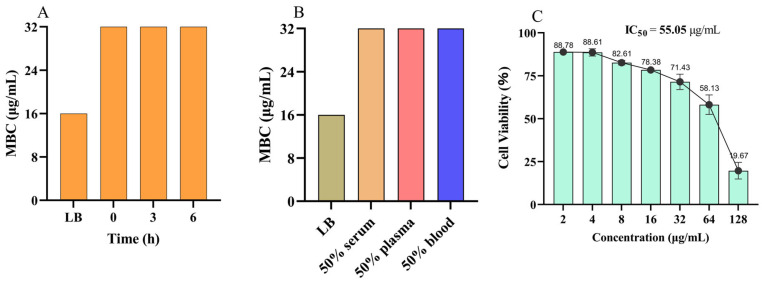
Plasma stability and cytotoxicity of compound **9h.** Plasma stability (**A**) and bactericidal activity in complex mammalian fluids (**B**), and in vitro cytotoxicity (**C**) of **9h** toward LO2 cells. Data are expressed as mean ± standard deviation (n = 3).

Due to the presence of various biological enzymes in plasma, natural AMPs are susceptible to enzymatic degradation upon entering the bloods, leading to a loss of their antimicrobial activity [43,44]. To evaluate the plasma stability of active compound **9h**, we conducted a stability assay by determining their MBC (minimum bactericidal concentration) values in plasma and different blood components. As shown in Figure 3A, the MBC value of **9h** dissolved in Mueller Hinton broth (MHB) against *S. aureus* ATCC4330 was 16 μg/mL. Subsequent treatment with 50% plasma resulted in an increase in the MBC value for **9h** to 32 μg/mL. Notably, this MBC value remained stable and unchanged after both 3 and 6 h. The observed initial increase in the MBC value, followed by remaining unchanged, could potentially be attributed to the initial binding of compound **9h** with plasma proteins, causing a reduction in its activity. Once this binding became saturated, the bactericidal activity of the compound remained stable, further indicating the good stability of compound **9h** in plasma. Additionally, compound **9h** was also found to retain its antibacterial activity in 50% serum (MBC = 32 μg/mL), 50% plasma (MBC = 32 μg/mL), and 50% blood (MBC = 32 μg/mL) against *S. aureus* ATCC4330 (Figure 3B). The above results demonstrated that compound **9h** had good stability, even in serum, plasma, and blood. 

### 2.4. In Vitro Cytotoxicity Evaluation

A key component in the development of antimicrobial agents for clinical application is the assessment of their potential toxicity to mammalian cells. To assess the cytotoxic impact, compound **9h** was chosen to undergo in vitro testing for its cytotoxic effects on LO2 cells. As shown in Figure 3C, at a concentration of 8 μg/mL, compound **9h** exhibited a low impact on LO2 cells, maintaining their viability by 82.61%. Furthermore, when the concentration of **9h** increased to 32 μg/mL, LO2 cells still maintained 71.43% viability. These findings underscored that compound **9h** (IC_50_ = 55.05 μg/mL) had high safety toward towards mammalian cells.

### 2.5. In Vitro Time-Kill Kinetics

**Figure 4:**
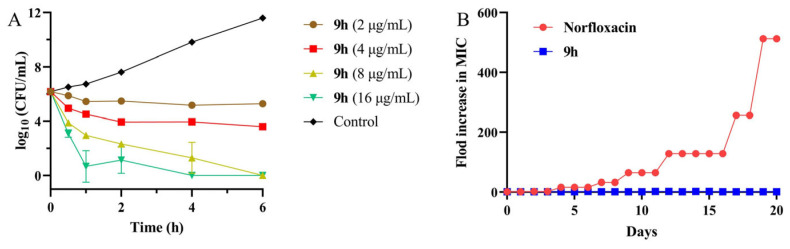
Time-kill kinetics (**A**) and bacterial resistance study (**B**) of **9h** against *S. aureus* ATCC43300. Data are expressed as mean ± standard deviation (n = 3).

Natural AMPs have rapid bactericidal properties [45], and in order to examine whether active compound **9h** have similar rapid bactericidal properties, the time-kill kinetics of **9h** were carried out to test the bactericidal action against *S. aureus* ATCC4330 at four different concentrations. As shown in Figure 4A, compound **9h** at low concentration (1×, 2× MIC) had a significant inhibitory effect on the growth of S. aureus ATCC43300, and the decreasing trend of bacterial load was obvious when compared to the negative control. As the concentrations increased, compound **9h** (4×, 8× MIC) exhibited rapid bactericidal activity. For example, compound **9h** (8× MIC, 16 μg/mL) achieved 5.95-log colony-forming unit (CFU) reduction (killing ≥ 99.9% of *S. aureus*) within 1 h compared with the control, and complete bacterial killing was observed after 4 h. These results demonstrated that compound **9h** had a strong bactericidal effect against Gram-positive bacteria. 

### 2.6. Resistance Development Studies

The rapid increase in bacterial resistance to antibiotics is a major challenge in clinical treatment [46], and in order to assess the drug resistance development for compound **9h**, we performed a drug resistance assay against *S. aureus* ATCC43300. Norfloxacin was selected as the positive control. The *S. aureus* cells were incubated with sub-MIC (1/2 MIC) concentration of compound **9h** and were serially passaged for a duration of 20 days. As shown in Figure 4B, the MIC value of **9h** against *S. aureus* ATCC43300 was almost consistent with its original value, and no more than a 4-fold increase was observed. In contrast, norfloxacin quickly induced resistance in *S. aureus*, showing a significant 512-fold increase in MIC value at the 19th passage. Thus, this result suggests that compound **9h** was unlikely to induce resistance in Gram-positive bacteria. 

### 2.7. Antibiofilm Activity Studies

**Figure 5:**
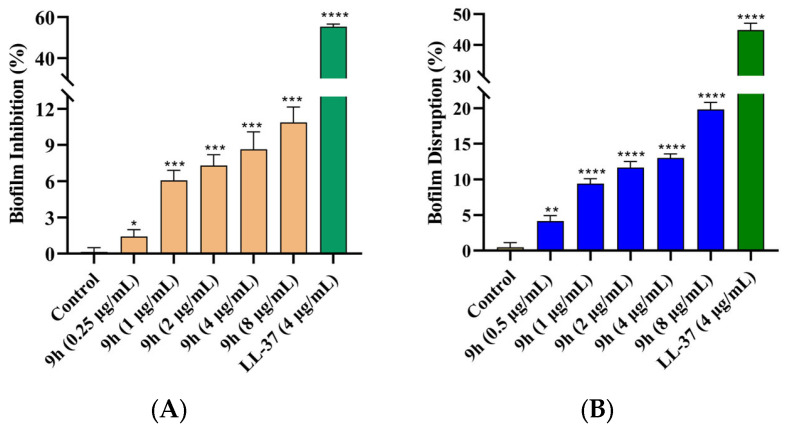
Antibiofilm activity of **9h**. (**A**) Inhibition of *S. aureus* biofilm formation by **9h**. (**B**) Eradication of the preformed *S. aureus* biofilm by **9h**. Error bars represent standard deviation from the mean of triplicate readout. * *p* < 0.05, ** *p* < 0.01, *** *p* < 0.001, and **** *p* < 0.0001, compared with the control group.

Bacterial biofilms created by the aggregation of bacteria is considered as one of the main reasons for the development of drug resistance [47]. In order to assess the ability of these xanthoangelol-derived compounds to inhibit or disrupt biofilms, the best compound, **9h**, was chosen against the biofilms of *S. aureus* ATCC43300. As illustrated in Figure 5A, compound **9h** was able to inhibit the biofilm formation of *S. aureus* in a concentration-dependent manner. Compound **9h** at 1× MIC (2 μg/mL) showed an 8.65% inhibition of *S. aureus* biofilm formation, which increased to 10.87% at 2× MIC, while the positive control LL-37 (4 μg/mL) displayed the inhibition of biofilm formation by 55.41%. Subsequently, we further assessed the efficiency of **9h** to eradicate the preformed biofilm. Matured *S. aureus* biofilm (grown for 24 h) was treated with compound **9h**, and the rate of biofilm disruption was then assessed by the crystal violet staining assay. As shown in Figure 5B, when treated with 1× MIC (2 μg/mL) of **9h**, only 11.65% of preformed *S. aureus* biofilm was eradicated, and about 19.83% of the eradication of the established *S. aureus* biofilm was achieved after treatment with 8 μg/mL of compound **9h**. These results suggest that compound **9h** had a relatively low ability to inhibit the biofilm formation and disrupt the preformed biofilm.

### 2.8. Antimicrobial Mechanism Studies

Since the derivatives developed here were designed based on mimicking AMPs, we hypothesized that they also acted through a membrane-interrupting mechanism similar to that of AMP. Given the excellent antimicrobial properties of compound **9h**, we chose it as the model molecule to further explore its antimicrobial mechanisms through a series of experiments, including fluorescence and scanning electron microscopy (SEM), membrane depolarization and permeability assays, ROS measurements, and DNA and protein leakage studies.

#### 2.8.1. Fluorescence and Electron Scanning Microscopy 

**Figure 6:**
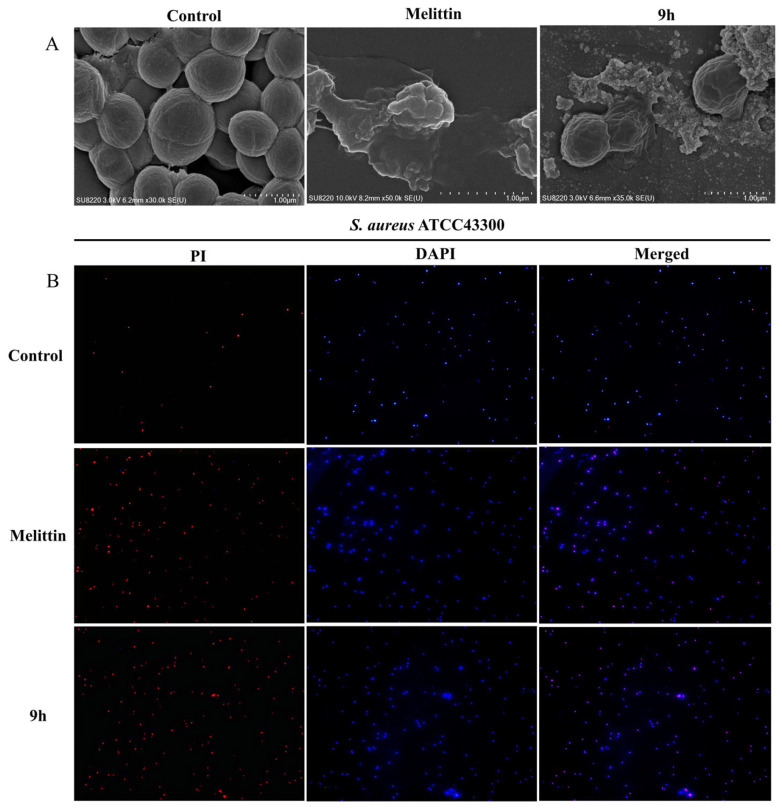
Fluorescence and electron scanning microscopy. (**A**) SEM images of the cell membrane of *S. aureus* cells, scar bar: 1.00 um. (**B**) Fluorescence micrographs of *S. aureus* cells stained with DAPI and PI and treated with **9h,** scar bar: 50 um.

Fluorescent dyes propidium iodide (PI) and 4′,6′-diamidino-2-phenylindole dihydrochloride (DAPI), as indicators of bacteria cell membrane damage, were used to perform fluorescence microscopy. DAPI was able to penetrate both living and dead bacterial cell membranes and bond with DNA to emit blue fluorescence, while PI can only pass through impaired cell membrane and integrates into double-stranded DNA to emit red fluorescence [48,49]. If the bacterial cell membrane is damaged, it leads to the emission of a significant quantity of red fluorescence. As shown in Figure 6B, the control group emitted distinct blue fluorescence and only very weak red fluorescence, suggesting that the vast majority of bacteria were living and their cell membranes were intact. By contrast, obvious red and blue fluorescence was observed at the same time after treatment with compounds **9h**, indicating that a lot of bacteria had died, and that the integrity of bacterial cell membranes had been disrupted. Furthermore, the surface changes in cell morphology of *S. aureus* ATCC43300 treated with compound **9h** were observed using SEM. The surface morphology of untreated *S. aureus* cells was intact and smooth. However, after treatment with compound **9h**, the *S. aureus* cells had lost their normal morphology, and they appeared wrinkled, collapsed, and ruptured (Figure 6A). This result directly demonstrated that compound **9h** could effectively disrupt the cell membranes of *S. aureus*.

#### 2.8.2. Cytoplasmic Membrane Depolarization

**Figure 7:**
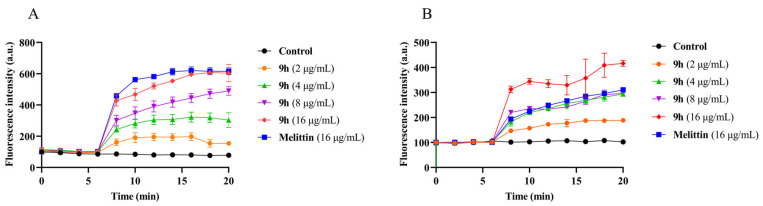
Antibacterial mechanism of compound **9h**. (**A**) Cytoplasmic membrane depolarization against *S. aureus* by compound **9h**. (**B**) Cell membrane permeabilization against *S. aureus* by compound **9h**. Data are expressed as mean ± standard deviation (n = 3).

To explore the antibacterial mechanism of compound **9h**, a cytoplasmic membrane depolarization assay was performed against *S. aureus* ATCC43300 using 3,3′-dipropylthiadicarbocyanine iodide (DiSC3(5)) assay. In general, DiSC3(5) could penetrate the intact cell membranes and was quenched when accumulated inside the bacterial cells. Once the cell membrane was destroyed, DiSC3(5) was released from the cell membrane, leading to a significant increase in fluorescence intensity [50,51]. As shown in Figure 7A, the fluorescence intensity of the blank control did not exhibit any significant variation, whereas a sharp enhancement in fluorescence intensity was observed after the addition of **9h** (1×, 2×, 4×, 8× MIC). Moreover, the fluorescence intensity in *S. aureus* increased in a dose-dependent manner with increasing concentrations of **9h**. It could be found that when treated with 16 μg/mL of **9h**, the fluorescence intensity (at 20 min) increased about 5-fold compared with that of the blank control group. The result thus suggested that compound **9h** had the ability to depolarize the membrane potential of cell membrane, thus increasing their permeability.

#### 2.8.3. Cell Membrane Permeabilization

The cell membrane permeabilization of compound **9h** against *S. aureus* ATCC43300 was assessed by PI uptake assay. PI could only pass through the impaired cell membrane and had been widely used to evaluate the cell membrane permeability. When the cell membranes were disrupted, PI entered into the cell membrane, leading to a significant improvement in fluorescence intensity. Therefore, the degree of membrane damage could be reflected by observing the fluorescence intensity in *S. aureus*. According to Figure 7B, the fluorescence intensity in the control group was weak and remained unchanged, whereas the fluorescence intensity of PI progressively increased when different concentrations of **9h** were added. Compared with the control group, the fluorescence intensity (at 20 min) increased approximately 5-fold after the addition of **9h** (16 μg/mL). The abovementioned results demonstrated that compound **9h** had a strong ability of cell membrane permeabilization in a concentration-dependent manner.

#### 2.8.4. Reactive Oxygen Species (ROS) Measurement

**Figure 8:**
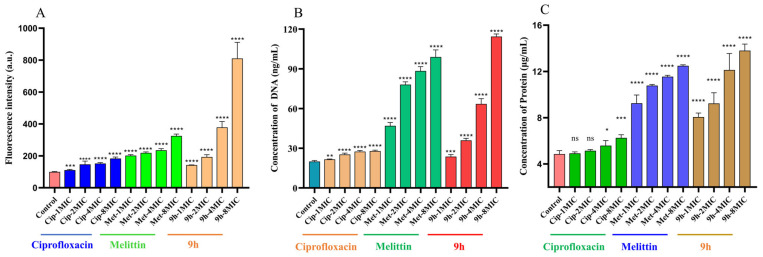
Antibacterial mechanism of compound **9h** against *S. aureus* ATCC43300. (**A**) Intracellular ROS changes after the treatment of **9h**. (**B**) DNA leakage caused by compound **9h**. (**C**) Protein leakage caused by compound **9h**. Data are expressed as mean ± standard deviation (n = 3). ns, not significant. * *p* < 0.05, ** *p* < 0.01, *** *p* < 0.001, and **** *p* < 0.0001, compared with the control group.

It is known that the disruption of bacterial cell membranes often causes the production of ROS in bacterial cells [31,52]. The fluorescent probe DCFH-DA was used to monitor the level of intracellular ROS produced by *S. aureus* ATCC43300. DCFH-DA is initially non-fluorescent; however, it undergoes hydrolysis to DCFH upon entry into the cell. Subsequently, DCFH is converted to the fluorescent compound DCF when ROS are produced in the cells. The non-membrane-targeted antibiotic ciprofloxacin (MIC = 0.25 μg/mL, MBC = 2 μg/mL against *S. aureus* ATCC43300), membrane-targeted peptide melittin (MIC = 8 μg/mL, MBC = 32 μg/mL against *S. aureus* ATCC43300), and PBS were used as controls. As demonstrated in Figure 8A, compound **9h** was able to effectively increase the level of ROS in *S. aureus* cells in a concentration-dependent manner. The fluorescence intensity of **9h** was about 8-fold higher than the PBS-treated group when the concentration of **9h** increased 16 μg/mL. Melittin was also able to dose-dependently increase the fluorescence intensity, whereas the ciprofloxacin-treated group showed little change in fluorescence intensity. The above result suggests that compound **9h** could disrupt bacterial cell membranes, thus inducing the production of ROS. 

#### 2.8.5. Leakage of DNA and Protein

The destruction of bacterial cell membranes usually causes the leakage of macromolecules, including DNA and proteins [53,54,55]. To investigate the effect of the compound **9h** on the leakage of cytoplasmic contents, we performed DNA and protein leakage assays against *S. aureus* ATCC43300. The degree of damage to the bacterial cell membrane could be reflected by the concentrations of DNA and protein. The non-membrane-targeted antibiotic ciprofloxacin, membrane-targeted peptide melittin, and PBS were used as controls. As depicted in Figure 8B, compound **9h** was able to cause the leakage of *S. aureus* contents (DNA) in a dose-dependent manner, exhibiting a similar action to that of melittin. The concentration of DNA excreted from *S. aureus* was significantly increased, and the concentration was approximately 5.5-fold higher than that of the blank control when the concentration of **9h** was 16 μg/mL. However, the DNA concentration in the ciprofloxacin-treated group changed little. A similar result were also observed in the leakage of protein with *S. aureus* (Figure 8C). These findings suggest that compound **9h** was able to disrupt the bacterial cell membrane, leading to the leakage of DNA and protein.

Altogether, the above results indicated that the xanthoangelol-derived compound **9h** acted through a membrane-interrupting mechanism and was able to destroy the integrity of bacterial cell membranes by disrupting the polarized state of the membrane and increasing membrane permeability, which is accompanied by intracellular ROS production, as well as the leakage of DNA and proteins, thus accelerating bacterial death.

## 3. Materials and Methods

### 3.1. Chemistry

All chemicals used in this study were of analytical grade and sourced from reputable commercial suppliers. The progress of reactions was monitored by analytical thin-layer chromatography (TLC) on silica gel GF254 plates. ^1^H NMR and ^13^C NMR spectra of all compounds were determined using a Bruker (Billerica, MA, USA) Avance instrument (500 MHz) with solvents CDCl_3_, DMSO-d_6_, or CD_3_OD. A Thermo (Waltham, MA, USA) DFS mass spectrometer was used to identify the HRMS of target compounds. The purity of all final compounds was verified to exceed 95% through HPLC analysis. 

#### 3.1.1. 1-(5-Hydroxy-2-methyl-2-(4-methylpent-3-en-1-yl)-2H-chromen-6-yl)ethan-1-one (**3**) 

To a stirred solution of compound **1** (10.00 g, 65.72 mmol) in pyridine (20.00 mL), we added citraldimethylacetal (26.06 g, 131.44 mmol), and the mixture was stirred at 150 °C for 10 h. After the reaction came to end, the solvent was evaporated in vacuo, and the crude product was purified by chromatography on silica gel column to give colorless oil: **3** (11.10 g, 59%). ^1^H NMR (500 MHz, CDCl_3_) δ 12.88 (s, 1H), 7.40 (d, *J* = 8.8 Hz, 1H), 6.66 (d, *J* = 10.1 Hz, 1H), 6.22 (d, *J* = 8.8 Hz, 1H), 5.43 (d, *J* = 10.2 Hz, 1H), 5.00 (t, *J* = 7.3 Hz, 1H), 2.43 (d, *J* = 1.5 Hz, 3H), 2.01 (h, *J* = 8.2 Hz, 2H), 1.70–1.54 (m, 5H), 1.45 (s, 3H), 1.32 (s, 3H). ^13^C NMR (125 MHz, CDCl_3_) δ 201.6, 159.0, 158.6, 130.8, 130.6, 126.0, 122.8, 115.2, 112.7, 107.9, 107.0, 79.2, 40.6, 26.1, 25.0, 24.6, 21.6, 16.6.

#### 3.1.2. 1-(5-(3-Bromopropoxy)-2-methyl-2-(4-methylpent-3-en-1-yl)-2H-chromen-6-yl)ethan-1-one (**4**)

To a stirred suspension of **3** (10.00 g, 34.92 mmol) in CH_3_CN (150 mL), we added K_2_CO_3_ (7.24 g, 52.38 mmol) and 1,3-dibromopropane (31.72 g, 157.14 mmol) slowly, and the reaction mixture was then stirred at 60 °C for 8 h. When the reaction was completed, the mixture was cooled to room temperature and extracted by ethyl acetate (40 mL × 3). The organic layer was dried, evaporated, and purified by flash column chromatography to give colorless oil: **4** (11.66 g, 82%). ^1^H NMR (500 MHz, CDCl_3_) δ 7.54 (d, *J* = 8.6 Hz, 1H), 6.66 (dd, *J* = 10.1, 0.8 Hz, 1H), 6.60 (dd, *J* = 8.6, 0.8 Hz, 1H), 5.65 (d, *J* = 10.1 Hz, 1H), 5.09 (dq, *J* = 7.1, 2.8, 1.3 Hz, 1H), 3.97 (t, *J* = 5.9 Hz, 2H), 3.67 (t, *J* = 6.4 Hz, 2H), 2.57 (s, 3H), 2.36 (p, *J* = 6.1 Hz, 2H), 2.16–2.04 (m, 2H), 1.81–1.63 (m, 5H), 1.57 (d, *J* = 1.3 Hz, 3H), 1.41 (s, 3H). ^13^C NMR (125 MHz, CDCl_3_) δ 197.9, 158.2, 155.0, 132.0, 131.2, 129.8, 125.2, 123.8, 117.0, 115.0, 112.4, 79.4, 73.2, 41.4, 33.2, 29.9, 29.8, 26.7, 25.6, 22.6, 17.6.

#### 3.1.3. General Procedure for the Preparation of **5a**–**5h**

To a solution of **4** (0.30 g, 0.74 mmol) in CH_3_CN (20 mL), we added K_2_CO_3_ (0.12 g, 0.89 mmol) and the corresponding amino (0.89 mmol), and the mixture was stirred at 60 °C for 8 h. After the reaction was completed, the solvents were removed, and the crude product was purified to afford **5a**–**5j**, yield 53–87%. 

##### 3-((6-Acetyl-2-methyl-2-(4-methylpent-3-en-1-yl)-2H-chromen-5-yl)oxy)-N,N,N-triethylpropan-1-aminium (**5a**)

Colorless oil, yield 67%. 1H NMR (500 MHz, CDCl_3_) δ 7.48 (d, *J* = 8.7 Hz, 1H), 6.59–6.50 (m, 2H), 5.63 (d, *J* = 10.2 Hz, 1H), 5.01 (dq, *J* = 8.6, 7.2, 1.5 Hz, 1H), 3.89 (t, *J* = 5.3 Hz, 2H), 3.66–3.61 (m, 2H), 3.55 (q, *J* = 7.2 Hz, 6H), 2.43 (s, 3H), 2.31 (dt, *J* = 11.3, 5.5 Hz, 2H), 2.01 (dq, *J* = 10.2, 5.9, 5.0 Hz, 2H), 1.72–1.54 (m, 5H), 1.49 (d, *J* = 1.4 Hz, 3H), 1.36 (dd, *J* = 13.6, 6.4 Hz, 12H). ^13^C NMR (125 MHz, CDCl_3_) δ 197.2, 158.5, 154.1, 132.0, 131.9, 130.5, 123.8, 123.7, 116.6, 115.7, 112.3, 79.8, 70.9, 53.6, 41.4, 29.3, 26.9, 25.7, 23.3, 22.6, 17.7, 8.0. HRMS (ESI) C_27_H_42_NO_3_ [M]^+^ calcd = 428.3159; found [M]^+^ = 428.3167.

##### 1-(5-(3-(Ethylamino)propoxy)-2-methyl-2-(4-methylpent-3-en-1-yl)-2H-chromen-6-yl)ethan-1-one (**5b**)

Colorless oil, yield 78%. ^1^H NMR (500 MHz, CDCl_3_) δ7.54 (d, *J* = 8.6 Hz, 1H), 6.61 (dd, *J* = 21.5, 9.4 Hz, 2H), 5.62 (d, *J* = 10.1 Hz, 1H), 5.08 (dt, *J* = 7.3, 5.8, 1.5 Hz, 1H), 3.90 (t, *J* = 6.3 Hz, 2H), 2.89 (t, *J* = 6.9 Hz, 2H), 2.74 (q, *J* = 7.1 Hz, 2H), 2.58 (s, 3H), 2.07 (dq, *J* = 23.8, 6.2 Hz, 4H), 1.78–1.73 (m, 1H), 1.69–1.62 (m, 4H), 1.56 (d, *J* = 1.3 Hz, 3H), 1.41 (s, 3H), 1.16 (t, *J* = 7.1 Hz, 3H). ^13^C NMR (125 MHz, CDCl_3_) δ 198.3, 158.3, 155.4, 132.0, 131.3, 129.6, 123.8, 117.1, 115.0, 112.4, 79.4, 74.5, 46.7, 44.2, 41.4, 30.0, 26.8, 25.7, 22.6, 17.6, 15.0. HRMS (ESI) C_23_H_34_NO_3_ [M + H]^+^ calcd = 372.2533; found [M + H]^+^ = 372.2538.

##### 1-(5-(3-(Diethylamino)propoxy)-2-methyl-2-(4-methylpent-3-en-1-yl)-2H-chromen-6-yl)ethan-1-one(**5c**) 

Colorless oil, yield 87%. ^1^H NMR (500 MHz, CDCl_3_) δ7.53 (d, *J* = 8.6 Hz, 1H), 6.66 (d, *J* = 10.1 Hz, 1H), 6.58 (d, *J* = 8.6 Hz, 1H), 5.62 (d, *J* = 10.1 Hz, 1H), 5.08 (tdd, *J* = 7.1, 3.1, 1.5 Hz, 1H), 3.87 (t, *J* = 6.5 Hz, 2H), 2.69 (q, *J* = 7.4 Hz, 2H), 2.59 (d, *J* = 6.4 Hz, 7H), 2.10 (tt, *J* = 11.9, 6.9 Hz, 2H), 2.00 (p, *J* = 6.9 Hz, 2H), 1.75 (dd, *J* = 14.0, 10.2, 6.4 Hz, 1H), 1.69–1.61 (m, 4H), 1.41 (s, 3H), 1.06 (t, *J* = 7.2 Hz, 6H). ^13^C NMR (125 MHz, CDCl_3_) δ 198.4, 158.2, 155.7, 131.9, 131.1, 129.4, 125.3, 123.8, 117.3, 114.9, 112.3, 79.3, 74.6, 49.5, 46.7, 41.4, 30.1, 27.6, 26.7, 25.7, 22.6, 17.6, 11.5. HRMS (ESI) C_25_H_38_NO_3_ [M + H]^+^ calcd = 400.2846; found [M + H]^+^ = 400.2847.

##### 1-(5-(3-(2,6-Dimethylmorpholino)propoxy)-2-methyl-2-(4-methylpent-3-en-1-yl)-2H-chromen-6-yl)ethan-1-one (**5d**)

Colorless oil, yield 82%. ^1^H NMR (500 MHz, CDCl_3_) δ7.54 (d, *J* = 8.6 Hz, 1H), 6.70 (d, *J* = 10.1 Hz, 1H), 6.59 (d, *J* = 8.6 Hz, 1H), 5.61 (d, *J* = 10.1 Hz, 1H), 5.15–5.02 (m, 1H), 3.89 (t, *J* = 6.3 Hz, 2H), 3.78–3.63 (m, 2H), 2.75 (d, *J* = 10.5 Hz, 2H), 2.59 (s, 3H), 2.54 (t, *J* = 7.2 Hz, 2H), 2.09 (dq, *J* = 11.8, 6.2 Hz, 2H), 2.01 (p, *J* = 6.6 Hz, 2H), 1.81–1.72 (m, 3H), 1.71–1.62 (m, 4H), 1.57 (s, 3H), 1.41 (s, 3H), 1.17 (d, *J* = 6.3 Hz, 6H). ^13^C NMR (125 MHz, CDCl_3_) δ198.3, 158.2, 155.6, 132.0, 131.1, 129.4, 125.3, 123.8, 117.3, 114.9, 112.4, 79.3, 74.0, 71.7, 59.5, 54.6, 45.9, 41.4, 30.2, 28.7, 27.0, 26.7, 25.7, 22.6, 19.2, 17.6. HRMS (ESI) C_27_H_40_NO_4_ [M + H]^+^ calcd = 442.2952; found [M + H]^+^ = 442.2958.

##### 1-(4-(3-((6-Acetyl-2-methyl-2-(4-methylpent-3-en-1-yl)-2H-chromen-5-yl)oxy)propyl)piperazin-1-yl)ethan-1-one (**5e**) 

Colorless oil, yield 75%. 1H NMR (500 MHz, CDCl_3_) δ7.53 (d, *J* = 8.6 Hz, 1H), 6.69 (d, *J* = 10.1 Hz, 1H), 6.59 (d, *J* = 8.6 Hz, 1H), 5.61 (d, *J* = 10.1 Hz, 1H), 5.08 (t, *J* = 6.5 Hz, 1H), 3.90 (t, *J* = 6.3 Hz, 2H), 3.68–3.59 (m, 2H), 3.52–3.43 (m, 2H), 2.59 (d, *J* = 4.7 Hz, 5H), 2.46 (dt, *J* = 16.4, 5.0 Hz, 4H), 2.10 (s, 5H), 2.01 (p, *J* = 6.6 Hz, 2H), 1.81–1.72 (m, 1H), 1.70–1.62 (m, 4H), 1.56 (s, 3H), 1.41 (s, 3H). ^13^C NMR (125 MHz, CDCl_3_) δ198.2, 168.9, 158.2, 155.5, 132.0, 131.1, 129.4, 125.2, 123.7, 117.2, 114.9, 112.4, 79.3, 73.8, 54.4, 53.2, 52.7, 46.3, 41.4, 30.1, 27.2, 26.7, 25.7, 22.6, 21.3, 17.6. HRMS (ESI) C_27_H_39_N_2_O_4_ [M + H]^+^ calcd = 455.2904; found [M + H]^+^ = 455.2909.

##### 1-(5-(3-((2-(Dimethylamino)ethyl)amino)propoxy)-2-methyl-2-(4-methylpent-3-en-1-yl)-2H-chromen-6-yl)ethan-1-one (**5f**) 

Colorless oil, yield 65%. ^1^H NMR (500 MHz, CDCl_3_) δ7.55 (d, *J* = 8.7 Hz, 1H), 6.62 (dd, *J* = 24.8, 9.4 Hz, 2H), 5.62 (d, *J* = 10.1 Hz, 1H), 5.08 (dt, *J* = 8.5, 7.1, 1.4 Hz, 1H), 3.91 (t, *J* = 6.2 Hz, 2H), 2.93 (t, *J* = 6.9 Hz, 2H), 2.78 (t, *J* = 6.1 Hz, 2H), 2.58 (s, 3H), 2.48 (t, *J* = 6.1 Hz, 2H), 2.21 (s, 6H), 2.07 (tt, *J* = 13.1, 6.5 Hz, 4H), 1.81–1.63 (m, 5H), 1.56 (d, *J* = 1.4 Hz, 3H), 1.41 (s, 3H). 13C NMR (125 MHz, CDCl_3_) δ198.2, 158.3, 155.4, 131.9, 131.3, 129.6, 124.9, 123.7, 117.1, 115.0, 112.3, 79.4, 74.4, 58.6, 47.2, 46.9, 45.5, 41.4, 30.1, 29.9, 26.7, 25.7, 22.6, 17.6. HRMS (ESI) C_25_H_39_N_2_O_4_ [M + H]^+^ calcd = 415.2955; found [M + H]^+^ = 415.2951.

##### 1-(5-(3-((3-(Dimethylamino)-2,2-dimethylpropyl)(methyl)amino)propoxy)-2-methyl-2-(4-methylpent-3-en-1-yl)-2H-chromen-6-yl)ethan-1-one (**5g**)

Colorless oil, yield 53%. ^1^H NMR (500 MHz, CDCl_3_) δ7.55 (d, *J* = 8.6 Hz, 1H), 6.66 (d, *J* = 10.1 Hz, 1H), 6.59 (d, *J* = 8.6 Hz, 1H), 5.62 (d, *J* = 10.1 Hz, 1H), 5.08 (t, *J* = 7.1 Hz, 1H), 3.91 (t, *J* = 6.3 Hz, 2H), 2.86 (t, *J* = 6.8 Hz, 2H), 2.59 (s, 3H), 2.50 (s, 2H), 2.27 (s, 6H), 2.18 (s, 2H), 2.10 (tt, *J* = 12.0, 7.4 Hz, 2H), 2.02 (p, *J* = 6.5 Hz, 2H), 1.82–1.62 (m, 5H), 1.56 (s, 3H), 1.41 (s, 3H), 0.92 (s, 6H). ^13^C NMR (125 MHz, CDCl_3_) δ198.3, 158.2, 155.6, 132.0, 131.1, 129.5, 123.8, 117.2, 114.9, 112.3, 79.3, 74.6, 69.5, 59.6, 48.7, 47.4, 41.4, 30.1, 26.7, 25.7, 24.9, 22.6, 17.6. HRMS (ESI) C_29_H_47_N_2_O_3_ [M + H]^+^ calcd = 471.3581; found [M + H]^+^ = 471.3580.

##### 1-(2-Methyl-2-(4-methylpent-3-en-1-yl)-5-(3-((3-(pyrrolidin-1-yl)propropoxy)-2H-chromen-6-yl)ethan-1-one (**5h**) 

Colorless oil, yield 56%. ^1^H NMR (500 MHz, CDCl_3_) δ7.55 (d, *J* = 8.6 Hz, 1H), 6.69–6.54 (m, 2H), 5.63 (d, *J* = 10.1 Hz, 1H), 5.08 (dp, *J* = 8.6, 5.7, 1.4 Hz, 1H), 3.89 (t, *J* = 6.2 Hz, 2H), 2.92 (t, *J* = 6.7 Hz, 2H), 2.80 (t, *J* = 6.9 Hz, 2H), 2.58 (s, 3H), 2.55–2.43 (m, 6H), 2.08 (dp, *J* = 23.6, 7.0, 6.5 Hz, 4H), 1.84–1.69 (m, 7H), 1.68–1.60 (m, 4H), 1.56 (d, *J* = 1.4 Hz, 3H), 1.41 (s, 3H). ^13^C NMR (125 MHz, CDCl_3_) δ198.1, 158.4, 155.3, 132.0, 131.4, 129.7, 124.7, 123.7, 117.0, 115.1, 112.4, 79.5, 74.4, 54.8, 54.2, 47.0, 41.4, 29.7, 26.8, 25.7, 23.4, 22.6, 17.6. HRMS (ESI) C_28_H_43_N_2_O_3_ [M + H]^+^ calcd = 455.3268; found [M + H]^+^ = 455.3272

#### 3.1.4. 1-(5-Hydroxy-2,8-dimethyl-2,8-bis(4-methylpent-3-en-1-yl)-2H,8H-pyrano[2,3-f]chromen-6-yl)ethan-1-one (7) 

To a solution of compound **6** (7.50 g, 49.29 mmol) in pyridine (20.00 mL), we added citraldimethylacetal (19.55 g, 98.58 mmol) slowly, and the resulting reaction mixture was stirred at 150 °C for 10 h. An excess of citraldimethylacetal (19.55 g, 98.58 mmol) was added, and the mixture was stirred for another 8 h. After the reaction, excess solvent was removed and extracted with ethyl acetate (25 × 3 mL). The combined organics were dried and concentrated under reduced pressure. The crude product was purified by silica gel column chromatography to afford a colorless oil: **7** (14.34 g, 50%). ^1^H NMR (500 MHz, CDCl_3_) δ 14.01 (s, 1H), 6.65 (dd, *J* = 33.6, 10.1 Hz, 2H), 5.49–5.29 (m, 2H), 5.09 (dd, *J* = 11.6, 5.8, 2.8, 1.4 Hz, 2H), 2.65 (s, 3H), 2.11 (dq, *J* = 24.3, 7.9, 7.1 Hz, 4H), 1.88–1.64 (m, 10H), 1.56 (d, *J* = 4.7 Hz, 6H), 1.45–1.40 (m, 6H). ^13^C NMR (125 MHz, CDCl_3_) δ 203.1, 160.6, 157.0, 155.3, 132.1, 131.8, 124.2, 123.90, 123.7, 123.3, 117.0, 116.6, 105.3, 102.0, 101.7, 81.0, 80.6, 41.7, 41.6, 33.2, 27.0, 26.9, 26.8, 25.7, 25.6, 23.1, 22.6, 17.6.

#### 3.1.5. (E)-1-(5-(3-Bromopropoxy)-8-(4-methoxypent-3-en-1-yl)-2,8-dimethyl-2-(4-methylpent-3-en-1-yl)-2H,8H-pyrano[2,3-f]chromen-6-yl)ethan-1-one (**8**)

The preparation method of **8** was the same as the general procedure depicted for **4**, colorless oil, yield 81%. ^1^H NMR (500 MHz, CDCl_3_) δ 6.64 (d, *J* = 10.1 Hz, 1H), 6.53 (d, *J* = 10.1 Hz, 1H), 5.54–5.45 (m, 2H), 5.09 (dtp, *J* = 7.2, 4.4, 1.4 Hz, 2H), 3.96 (tt, *J* = 5.8, 3.2 Hz, 2H), 3.60 (t, *J* = 6.5 Hz, 2H), 2.50 (s, 3H), 2.29–2.20 (m, 2H), 2.08 (tt, *J* = 16.5, 6.9 Hz, 4H), 1.78–1.65 (m, 10H), 1.57 (d, *J* = 2.9 Hz, 6H), 1.42–1.33 (m, 6H). ^13^C NMR (125 MHz, CDCl_3_) δ 2007, 152.2, 151.8, 150.7, 131.9, 127.0, 126.6, 123.9, 123.8, 117.3, 117.1, 117.1, 116.7, 108.2, 108.1, 106.2, 106.2, 79.72, 79.2, 73.5, 41.5, 41.1, 33.1, 32.8, 30.2, 26.5, 26.4, 26.3, 25.7, 22.8, 22.6, 22.6, 17.6.

#### 3.1.6. General Procedure for the Preparation of **9a**–**9h**

The target compound **9a**–**9h** was synthesized according to the procedure depicted for **5a**–**5h**, starting from intermediate **8**, yield 52–89%. 

##### 3-((6-Acetyl-2,8-dimethyl-2,8-bis(4-methylpent-3-en-1-yl)-2H,8H-pyrano[2,3-f]chromen-5-yl)oxy)-N,N,N-triethylpropan-1-aminium (**9a**)

Colorless oil, yield 84%. ^1^H NMR (500 MHz, CDCl_3_) δ 6.64 (d, *J* = 10.1 Hz, 1H), 6.45 (d, *J* = 10.0 Hz, 1H), 5.59–5.45 (m, 2H), 5.13–5.01 (m, 2H), 3.96 (dt, *J* = 7.1, 4.7 Hz, 2H), 3.59 (q, *J* = 7.2 Hz, 6H), 3.53–3.47 (m, 2H), 2.50 (s, 3H), 2.27 (dq, *J* = 11.0, 5.1 Hz, 2H), 2.06 (ddt, *J* = 21.7, 16.6, 8.4 Hz, 4H), 1.83–1.62 (m, 10H), 1.48–1.35 (m, 15H). ^13^C NMR (125 MHz, CDCl_3_) δ 200.8, 152.5, 151.8, 151.3, 132.0, 131 9, 127.6, 126.6, 123. 8, 123.7, 116.7, 116.6, 116.5, 108.2, 106.4, 80.1, 79.6, 79.5, 71.9, 55.1, 53.6, 41.5, 41.1, 32.9, 26.5, 26.4, 26.3, 25.6, 23.1, 22.9, 22.6, 22.5, 17.6, 7.9. HRMS (ESI) C_37_H_56_NO_4_ [M]^+^ calcd = 578.4204; found [M]^+^ = 578.4212.

##### 1-(3-((6-Acetyl-2,8-dimethyl-2,8-bis(4-methylpent-3-en-1-yl)-2H,8H-pyrano[2,3-f]chromen-5-yl)oxy)propyl)pyridin-1-ium (**9b**) 

Colorless oil, yield 89%.^1^H NMR (500 MHz, CDCl_3_) δ 9.76–9.62 (m, 2H), 8.56 (tt, *J* = 7.8, 1.4 Hz, 1H), 8.20–8.06 (m, 2H), 6.63 (d, *J* = 10.1 Hz, 1H), 6.37 (d, *J* = 10.0 Hz, 1H), 5.59–5.44 (m, 2H), 5.17 (hept, *J* = 6.6 Hz, 2H), 5.12–5.00 (m, 2H), 3.96 (dq, *J* = 5.7, 3.0, 2.4 Hz, 2H), 2.60 (p, *J* = 6.1 Hz, 2H), 2.52 (s, 3H), 2.18–1.99 (m, 4H), 1.81–1.64 (m, 10H), 1.59–1.54 (m, 6H), 1.42–1.35 (m, 6H). ^13^C NMR (125 MHz, CDCl_3_) δ 201.2, 152.4, 152.1, 151.3, 145.7, 145.2, 132.00, 131.9, 128.3, 127.5, 126.6, 123.7, 123.7, 116.8, 116.5, 107.9, 106.2, 80.1, 79.5, 79.4, 71.8, 59.3, 53.5, 41.5, 41.0, 33.1, 32.2, 26.5, 26.3, 26.2, 25.6, 25.6, 22.9, 22.6, 17.6. HRMS (ESI) C_36_H_46_NO_4_ [M]^+^ calcd = 556.3421; found [M]^+^ = 556.3430.

##### 1-(5-(3-(Diethylamino)propoxy)-2,8-dimethyl-2,8-bis(4-methylpent-3-en-1-yl)-2H,8H-pyrano[2,3-f]chromen-6-yl)ethan-1-one (**9c**)

Colorless oil, yield 63%. ^1^H NMR (500 MHz, CDCl_3_) δ6.63 (d, *J* = 10.1 Hz, 1H), 6.52 (d, *J* = 10.0 Hz, 1H), 5.51–5.44 (m, 2H), 5.08 (dt, *J* = 6.9, 3.5 Hz, 2H), 3.86 (td, *J* = 6.3, 3.3 Hz, 2H), 2.65–2.56 (m, 6H), 2.50 (s, 3H), 2.09 (t, *J* = 15.0 Hz, 4H), 1.90 (dt, *J* = 13.8, 6.4 Hz, 2H), 1.79–1.63 (m, 10H), 1.57 (s, 6H), 1.42–1.35 (m, 6H), 1.06 (t, *J* = 7.2 Hz, 6H). ^13^C NMR (125 MHz, CDCl_3_) δ 200.9, 152.7, 151.6, 150.6, 131.9, 131.8, 126.7, 126.5, 123.9, 123.9, 117.4, 116.7, 108.1, 108.1, 106.0, 106.0, 79.6, 79.1, 79.1, 74.7, 49.4, 46.7, 41.4, 41.1, 41.0, 32.7, 27.3, 26.4, 26.3, 26.3, 25.7, 22.8, 22.6, 22.6, 17.6, 11.5. HRMS (ESI) C_35_H_52_NO_4_ [M + H]^+^ calcd = 550.3891; found [M + H]^+^ = 550.3896.

##### 1-(5-(3-(2,6-Dimethylmorpholino)propoxy)-2,8-dimethyl-2,8-bis(4-methylpent-3-en-1-yl)-2H,8H-pyrano[2,3-f]chromen-6-yl)ethan-1-one (**9d**)

Colorless oil, yield 86%. ^1^H NMR (500 MHz, CDCl_3_) δ6.63 (d, *J* = 10.1 Hz, 1H), 6.55 (d, *J* = 10.0 Hz, 1H), 5.51–5.43 (m, 2H), 5.08 (dt, *J* = 7.2, 3.1, 1.5 Hz, 2H), 3.91–3.82 (m, 2H), 3.67 (dtd, *J* = 12.4, 6.1, 2.0 Hz, 2H), 2.95 (s, 1H), 2.88 (s, 1H), 2.78–2.73 (m, 2H), 2.49 (d, *J* = 10.8 Hz, 5H), 2.09 (tq, *J* = 15.9, 9.4, 8.6 Hz, 4H), 1.94–1.86 (m, 2H), 1.74 (dt, *J* = 15.8, 11.9, 5.4 Hz, 4H), 1.66 (d, *J* = 2.0 Hz, 6H), 1.42–1.36 (m, 6H), 1.17 (d, *J* = 6.3 Hz, 6H). ^13^C NMR (125 MHz, CDCl_3_) δ200.7, 162.5, 152.6, 151.6, 150.6, 131.8, 126.6, 126.5, 123.9, 123.9, 123.9, 117.4, 116.7, 108.1, 106.0, 79.6, 79.1, 74.3, 71.7, 59.5, 54.7, 414, 41.1, 41.0, 36.4, 31.4, 27.0, 26.3, 26.3, 25.7, 22.8, 22.6, 22. 6, 19.2, 17.6, 17.6. HRMS (ESI) C_37_H_54_NO_5_ [M + H]^+^ calcd = 592.3997; found [M + H]^+^ = 592.4001.

##### 2-(3-((6-Acetyl-2,8-dimethyl-2,8-bis(4-methylpent-3-en-1-yl)-2H,8H-pyrano[2,3-f]chromen-5-yl)oxy)propyl)isothiouronium (**9e**)

Colorless oil, yield 71%. ^1^H NMR (500 MHz, CDCl_3_) δ 9.09–7.12 (m, 4H), 6.63 (d, *J* = 10.1 Hz, 1H), 6.46 (d, *J* = 10.0 Hz, 1H), 5.59–5.42 (m, 2H), 5.16–5.01 (m, 2H), 3.94 (tq, *J* = 9.0, 5.2, 4.4 Hz, 2H), 3.43 (t, *J* = 7.2 Hz, 2H), 2.54 (s, 3H), 2.21–2.00 (m, 6H), 1.83–1.62 (m, 10H), 1.57 (s, 6H), 1.42–1.35 (m, 6H). ^13^C NMR (125 MHz, CDCl_3_) δ 201.8, 152.4, 152.2, 151.3, 132.0, 131.9, 127.5, 127.4, 126.6, 123.9, 123.7, 117.0, 116.57, 108.2, 106.3, 80.0, 79.5, 79.5, 73.5, 41.5, 41.1, 33.0, 29.5, 28.1, 26.5, 26.4, 26.3, 25.7, 22.9, 22.6, 22.6, 17.7. HRMS (ESI) C_32_H_45_N2O_4_S [M +H]^+^ calcd = 553.3095; found [M + H]^+^ = 553.3104.

##### 1-(2,8-Dimethyl-5-(3-(methyl(2-(methylamino)ethyl)amino)propoxy)-2,8-bis(4-methylpent-3-en-1-yl)-2H,8H-pyrano[2,3-f]chromen-6-yl)ethan-1-one (**9f**)

Colorless oil, yield 52%. ^1^H NMR (500 MHz, CDCl_3_) δ 6.63 (d, *J* = 10.1 Hz, 1H), 6.49 (d, *J* = 10.0 Hz, 1H), 5.55–5.43 (m, 2H), 5.09 (dtt, *J* = 8.7, 5.2, 1.7 Hz, 2H), 3.98–3.81 (m, 2H), 2.91 (t, *J* = 6.7 Hz, 2H), 2.83 (t, *J* = 6.2 Hz, 2H), 2.51 (d, *J* = 9.7 Hz, 5H), 2.24 (s, 6H), 2.04 (dtd, *J* = 38.9, 12.1, 11.1, 7.0 Hz, 6H), 1.82–1.61 (m, 10H), 1.57 (s, 6H), 1.4–1.33 (m, 6H). ^13^C NMR (125 MHz, CDCl_3_) δ 200.7, 152.6, 151.1, 131.9, 131.9, 127.0, 126.5, 123.9, 123.8, 117.1, 116.7, 108.2, 106.2, 79.8, 79.4, 744, 46.9, 46.8, 45.5, 41.5, 41.1, 32.9, 26.5, 26.4, 25.7, 22.9, 22.6, 17.6. HRMS (ESI) C_35_H_52_N_2_O_4_ [M + H]^+^ calcd = 565.4000; found [M + H]^+^ = 565.4004.

##### 1-(5-(3-((3-(Diethylamino)propyl)amino)propoxy)-2,8-dimethyl-2,8-bis(4-methylpent-3-en-1-yl)-2H,8H-pyrano[2,3-f]chromen-6-yl)ethan-1-one (**9g**)

Colorless oil, yield 72%. ^1^H NMR (500 MHz, CDCl_3_) δ6.63 (d, *J* = 10.1 Hz, 1H), 6.49 (d, *J* = 10.0 Hz, 1H), 5.51–5.44 (m, 2H), 5.08 (tt, *J* = 9.1, 7.4, 3.3 Hz, 2H), 3.88 (q, *J* = 5.7 Hz, 2H), 2.80 (t, *J* = 6.9 Hz, 2H), 2.71 (t, *J* = 6.9 Hz, 2H), 2.58–2.43 (m, 9H), 2.07 (tq, *J* = 14.8, 8.2, 7.3 Hz, 4H), 1.95 (h, *J* = 6.6 Hz, 2H), 1.81–1.63 (m, 12H), 1.39 (d, *J* = 6.7 Hz, 6H), 1.02 (t, *J* = 7.2 Hz, 6H). ^13^C NMR (125 MHz, CDCl_3_) δ200.8, 152.5, 151.8, 150.7, 131.9, 126.8, 126.5, 123.9, 117.3, 116.7, 108.1, 106.1, 79.7, 79.1, 74.5, 51.3, 48.8, 46.7, 41.4, 41.0, 32.7, 30.0, 26.7, 26.4, 26.3, 26.3, 25.6, 22.8, 22.6, 22.6, 17.6, 11.5. HRMS (ESI) C_38_H_59_N_2_O_4_ [M + H]^+^ calcd = 607.4469; found [M + H]^+^ = 607.4476.

##### 1-(2,8-Dimethyl-2,8-bis(4-methylpent-3-en-1-yl)-5-(3-((3-(4-methylpiperazin-1-yl)propyl)amino)propoxy)-2H,8H-pyrano [2,3-f]chromen-6-yl)ethan-1-one (**9h**)

Colorless oil, yield 77%. ^1^H NMR (500 MHz, CDCl_3_) δ6.64 (d, *J* = 10.1 Hz, 1H), 6.49 (d, *J* = 10.1 Hz, 1H), 5.57–5.43 (m, 2H), 5.08 (dt, *J* = 7.5, 5.9, 3.8, 1.8 Hz, 2H), 3.89 (dtd, *J* = 7.9, 6.0, 5.3, 2.6 Hz, 2H), 2.90 (dt, *J* = 11.0, 6.6 Hz, 4H), 2.51 (s, 3H), 2.47 (t, *J* = 6.8 Hz, 3H), 2.23 (s, 3H), 2.07 (qd, *J* = 11.8, 10.6, 4.8 Hz, 6H), 1.85 (p, *J* = 6.8 Hz, 2H), 1.80–1.62 (m, 10H), 1.57 (s, 6H), 1.43–1.35 (m, 6H). ^13^C NMR (125 MHz, CDCl_3_) δ 200.7, 152.4, 151.1, 132.0, 131.9, 127.1, 126.6, 123.8, 123.7, 117.0, 116.7, 116.6, 108.2, 106.2, 79.9, 79.4, 79.4, 74.2, 57.0, 55.0, 53.1, 48.9, 46.8, 45.9, 41.4, 41.1, 41.1, 32.9, 28.9, 26.4, 26.4, 25.6, 25.3, 22.8, 22.6, 17.6. HRMS (ESI) C_38_H_58_N_3_O_4_ [M + H]^+^ calcd = 620.4422; found [M + H]^+^ = 634.4594.

### 3.2. Biological Evaluation

#### 3.2.1. In Vitro Antibacterial Activity Assay

MICs of the derivatives were determined by the micro broth dilution method according to the American Clinical and Laboratory Standards Institute (CLSI) standards. Briefly, 2-fold serial dilutions of target compounds were prepared in 96-well plates containing TSB broth. An equal volume of bacterial suspension (10^6^ CFU/mL) was then pipetted into each well, and the mixture was incubated at 37 °C for 24 h. The MICs were defined as the lowest concentration of compounds tested that completely inhibited the visible growth of bacteria. All biological experiments were repeated three times.

#### 3.2.2. Hemolysis Assay

Sheep blood was used to isolate red blood cells (RBCs), which were then resuspended in 1× PBS (5%). The compounds were dissolved in PBS to give an initial concentration of 5.12 mg/mL, compound solution (100 μL) was added to the first well of a 96-well plate, two-fold serial dilutions of compounds were prepared in 96-well plates, and then 150 μL of 5% RBC suspension was added to each well. This was followed by incubation at 37 °C for 1 h. After incubation, the plate was centrifuged (3500 rpm, 5 min), and 100 μL of supernatant solution from each well was carefully pipetted into a new plate. The absorbance values were measured at 540 nm. Erythrocyte hemolysis rate = (Absorbance_compound_ – Absorbance _negative control_)/(Absorbance_1% Triton X-100_ − Absorbance _negative control_) × 100%. All biological experiments were repeated three times.

#### 3.2.3. Bactericidal Activity in Plasma and Complex Mammalian Fluids 

Compounds (1×, 2×, 4×, 8×, and 16× MIC) were mixed with 50% plasma, and the mixture was preincubated at 37 °C for 0, 3, and 6 h. MBCs of the compounds were tested. Furthermore, MBCs of compounds in 50% plasma, 50% serum, and 50% blood suspension were tested. The MBC value is defined as the concentration at which the colony count is less than 5 after 18 h of incubation at 37 °C. All biological experiments were repeated three times.

#### 3.2.4. Bactericidal Time-Kill Assay

*S. aureus* ATCC43300 was cultured at 37 °C for 6 h and diluted to 1×10^6^ CFU/mL. Next, the bacterial suspension was added with compound **9h** (1×, 2×, 4×, 8×, and 16× MIC) and then cultured at 37 °C (200 rpm) for 0, 0.5, 1, 2, 4, and 6 h. At each time point, the bacterial suspension (20 μL) was diluted in PBS, and the number of bacterial colonies was measured. All biological experiments were repeated three times.

#### 3.2.5. Resistance Development Assay

Resistance was defined as a more than 4-fold increase in the tested compound in MIC when compared to the beginning value. Briefly, *S. aureus* ATCC43300 was cultured (37 °C, 200 rpm) in MHB broth for 5 h and diluted to 1 × 10^6^ CFU/mL. Then, the bacteria were cultured with compound **9h** at a sub-MIC concentration (1/2 MIC) for 18 h. The MICs were determined according to this method for consecutive 20 days. All biological experiments were repeated three times.

#### 3.2.6. Biofilm Inhibition and Disruption Assay

Biofilm inhibition assay: *S. aureus* ATCC43300 was cultured (37 °C, 200 rpm) in MHB broth for 5 h and diluted to 1 × 10^6^ CFU/mL. Bacteria suspension (100 µL) was incubated with compound **9h** (1×, 2×, 4×, 8×, and 16× MIC, 100 µL) for 24 h at 37 °C. The planktonic supernatant cells were discarded and washed with PBS three times. Then, methanol (150 µL) was added to each well for 30 min. After discarding methanol, 0.1% crystal violet solution (150 µL) was added, and biofilms were stained for 15 min. Next, crystal violet solution was removed and washed with PBS three times. Finally, ethanol (150 µL) was pipetted into the wells, and the OD values were measured at 575 nm. The biofilm inhibition (%) = (OD_compound_ − OD_blank_)/(OD_positive control_ − OD_blank_) × 100%.

Biofilm disruption: Briefly, *S. aureus* ATCC43300 (1 × 10^6^ CFU/mL) was incubated in 96-well plates for 24 h to create biofilms. Next, the planktonic supernatant cells were removed. Following this, compound **9h** solution (1, 2, 4, 8, 16, 32× MIC) was added, and the plates were incubated at 37 °C for 24 h. Then, the treated biofilm was determined by crystal violet assay. The OD values were measured at 575 nm. Biofilm disruption (%) = (OD_compound_ − OD_blank_)/(OD_positive control_ − OD_blank_) × 100%. All biological experiments were repeated three times.

#### 3.2.7. Membrane Depolarization Assay

Mid-log *S. aureus* ATCC43300 was collected and then washed with PBS three times. Bacterial suspension (1.0 × 10^8^ CFU/mL, 150 μL) was added to a black 96-well plate, and then fluorescent dye DiSC3(5) (10 μM, 50 μL) was pipetted into each well of plates and preincubated in the dark for 30 min. After the incubation, the fluorescence intensity was measured every minute for 6 min using a microplate reader (SpectraMax i3x, Molecular Devices, Sunnyvale, CA, USA) (λex/λem = 622 nm/670 nm). Then, compound **9h** (1×, 2×, 4×, 8×, 16× MIC) was added to the wells, The fluorescence intensity was detected for the next 14 min. All biological experiments were repeated three times.

#### 3.2.8. Cell Membrane Permeabilization Assay

Mid-log *S. aureus* ATCC43300 was collected and then washed with PBS three times. Bacterial suspension (1.0 × 10^8^ CFU/mL, 150 μL) was added to a black 96-well plate; following this, fluorescent dye PI (10 μM, 50 μL) was added to each well of plates, and they were preincubated in the dark for 30 min. After the incubation, the fluorescence intensity was detected every minute for 6 min using a microplate reader (λex/λem = 535 nm/615 nm). Then, compound **9h** with different concentrations (1×, 2×, 4×, 8×, 16× MIC) was added to the wells. The fluorescence intensity was determined similarly for another 14 min. All biological experiments were repeated three times.

#### 3.2.9. DAPI/PI Fluorescence Assay

Mid-log *S. aureus* ATCC43300 was incubated with compound **9h** (4× MIC**)** for 2 h. Next, the mixture was centrifuged (4000 rpm, 5 min), and the supernatant was discarded and resuspended in 160 μL PBS (1 × 10^8^ CFU/mL). After incubation, PI (10 μg/mL, 20 μL) and DAPI (10 µg/mL, 20 μL) were added into the bacterial suspension, and the suspension was further cultured at 4 °C in the dark for 30 min. A total of 20 µL of the suspension was taken out, pipetted on slides, and recorded using a laser confocal microscope (Axio Vert AI, Carl Zeiss AG, Oberkochen, Germany). 

#### 3.2.10. Scanning Electron Microscopy (SEM)

*S. aureus* ATCC43300 (1 × 10^8^ CFU/mL) was incubated with compound **9h** (2× MIC) at 37 °C for 6 h. Next, the mixture was centrifuged (3000 rpm, 15 min), and the planktonic supernatant cells were discarded and washed with PBS for three times. The bacterial precipitate was stained with glutaraldehyde (2.5%) and dried by ethanol. Finally, the images were captured with an electron microscope (EPMA-1610, Shimadzu, Kyoto, Japan).

#### 3.2.11. ROS Assay

Mid-log *S. aureus* ATCC43300 was collected and resuspended in PBS. Bacteria suspension (1.0 × 10^8^ CFU/mL) was placed into a 96-well plate, and an equal volume of DCF-DA (10.0 μM) was mixed and incubated at 37 °C for 30 min. After incubation, the suspension was centrifuged (3500 rpm, 4 min), and the supernatant was removed and resuspended in PBS. Then, bacteria suspension was pipetted into a black 96-well plate and treated with **9h** (1×, 2×, 4×, 8× and 16× MIC) for 1 h in the dark. The fluorescence intensity of each well was recorded by a microplate reader (Ex = 488 nm and Em = 530 nm). All biological experiments were repeated three times.

#### 3.2.12. Protein and DNA Leakage Study

Mid-log *S. aureus* ATCC43300 was collected and resuspended in PBS (1.0 × 10^8^ CFU/mL). Compound **9h** (1×, 2×, 4×, 8×, and 16× MIC) was added to the bacteria suspension, and the mixture was cultured or another 4 h at 37 °C, followed by centrifugation (3500 rpm, 4 min). The concentration of the protein was determined according to the method of the BCA assay kit (Thermo Fisher Scientific, Waltham, MA, USA), and the concentration of DNA in the supernatant was tested by a micro-spectrophotometer. All biological experiments were repeated three times.

## 4. Conclusions

In summary, a series of novel amphiphilic xanthoangelol-derived compounds were designed and synthesized by mimicking the structure and function of AMPs. The in vitro bioactivity evaluation revealed that compound **9h** not only displayed excellent antibacterial activity against Gram-positive strains tested with MICs ranging from 0.5 to 2 μg/mL, comparable to vancomycin, but also had poor hemolytic activity (HC_50_ = 98.0 μg/mL) and good selectivity (SI = 49.0). Additionally, the rapid bactericidal effects, low risk of drug resistance development, low cytotoxicity, and good plasma stability were additional advantages of compound **9h**. Mechanistic studies revealed that compound **9h** had good membrane-interrupting capabilities and could disrupt the integrity of bacterial cell membranes by destroying the polarized state of the membrane and increasing membrane permeability, causing an increase in intracellular ROS and the leakage of DNA and proteins, thus accelerating bacterial death. Taken together, these findings indicate that compound **9h** showed promise for further development as a novel antimicrobial agent to combat infections caused by Gram-positive bacteria. 

## Figures and Tables

**Table 1:** Antibacterial activity (MIC, μg/mL) against Gram-positive bacteria and hemolytic activity of compounds **5a**–**5h** and **9a**–**9h**.

Compd.	Gram-Positive Bacteria								HC_50_	SI
	*S. a.* 25923	*S. a.* 31007	*S. a* 43300	*B. s.* 9372	*E. f.* 29212	*S. e.* 12228	*E. f.* 51299	*E. f.* 51559		
**5a**	8	128	64	64	64	64	16	16	571.5	8.9
**5b**	16	16	16	8	8	8	32	32	118.4	7.4
**5c**	32	32	32	16	32	16	128	64	324.1	10.1
**5d**	>128	>128	>128	>128	>128	>128	>128	>128	746.5	<5.8
**5e**	128	128	128	64	32	64	128	128	62.3	0.49
**5f**	16	16	8	8	8	8	32	32	127.7	16.0
**5g**	8	8	8	8	8	4	32	16	183.1	22.9
**5h**	8	8	8	8	8	4	32	32	84.7	4.3
**9a**	2	4	4	1	4	2	4	4	40.4	10.1
**9b**	2	4	1	2	2	32	4	4	26.6	26.6
**9c**	2	2	4	2	1	1	8	4	187.6	46.9
**9d**	>128	>128	>128	>128	>128	>128	>128	>128	>1280	>10
**9e**	8	128	64	64	64	128	>128	>128	>1280	>20
**9f**	4	4	4	8	8	8	4	8	40.3	10.1
**9g**	2	4	4	2	1	1	32	32	34.9	8.7
**9h**	2	2	2	2	1	0.5	8	4	98.0	49.0
**Van**	1	1	2	0.5	2	2	128	128	ND	ND
**Cos**	128	128	128	16	128	64	>128	>128	ND	ND

*S. a. *25923: *Staphylococcus aureus* ATCC25923, erythromycin-susceptible strain; *S. a.* 31007: *Staphylococcus aureus* ATCC31007, penicillin-resistant strain; *S. a.* 43300: *Staphylococcus aureus* ATCC43300, methicillin-resistant strain; *B. s.* 9372; *Bacillus subtilis* ATCC9372, penicillin-susceptible strain; *E. f.* 29212: *Enterococcus faecalis* ATCC29212, vancomycin-susceptible strain; *S. e.* 12228: *Staphylococcus epidermidis* ATCC12228, erythromycin-susceptible strain; *E. f.* 51299: *Enterococcus faecalis* ATCC51299, vancomycin-resistant strain; *E. f* 51559: *Enterococcus faecium* ATCC51559, vancomycin-resistant strain; Van = vancomycin; Cos = colistin; ND: not determined.

**Table 2:** Antibacterial activity (MIC, μg/mL) against Gram-negative bacteria of compounds **5a**–**5h** and **9a**–**9h**.

Compd.	Gram-Negative Bacteria			
	*K. p.* 10031	*A. b.* 19606	*E. c*. 25922	*P. a.* 27853
**5a**	128	>128	>128	>128
**5b**	16	16	32	>128
**5c**	16	32	128	32
**5d**	128	128	>128	>128
**5e**	>128	128	>128	>128
**5f**	8	8	8	32
**5g**	4	8	16	128
**5h**	8	4	8	16
**9a**	>128	128	>128	>128
**9b**	32	32	128	>128
**9c**	>128	>128	>128	>128
**9d**	>128	>128	>128	>128
**9e**	>128	>128	>128	>128
**9f**	16	64	128	>128
**9g**	8	4	4	32
**9h**	8	4	4	16
**Van**	128	16	128	>128
**Cos**	1	1	0.5	0.5

*K. p.* 10031: *Klebsiella pneumonia* ATCC10031; *A. b.* 19606: *Acinetobacter baumannii* ATCC19606; *E. c.* 25922: *Escherichia coli* ATCC25922, penicillin-susceptible strain; *P. a.* 27853: *Pseudomonas aeruginosa* ATCC27853, penicillin-susceptible strain. Van = vancomycin; Cos = colistin.

## Data Availability

Data are contained within the article and Supplementary Materials.

## Supplementary Materials

The supporting information can be downloaded at https://www.mdpi.com/article/10.3390/antibiotics13080744/s1. File S1. ^1^H NMR, ^13^C NMR Spectra and HRMS of target compounds.
